# Study on the Damage Mechanisms in the Forming Process of High-Strength Steel Laser Tailor Welded Blanks Based on the Johnson–Cook Damage Model

**DOI:** 10.3390/ma18153497

**Published:** 2025-07-25

**Authors:** Xianping Sun, Huaqiang Li, Song Gao, Qihan Li

**Affiliations:** School of Mechatronic Engineering, Changchun University of Technology, Changchun 130012, China; lihuaqiang0221@163.com (H.L.); gaosong@ccut.edu.cn (S.G.); liqihan@ccut.edu.cn (Q.L.)

**Keywords:** tailor welded blanks (TWBs), J–C damage model, stress triaxiality, fracture strain, forming limit diagram (FLD)

## Abstract

This paper, based on the Johnson–Cook damage model, investigates the damage mechanism of high-strength steel tailor welded blanks (TWBs) (Usibor1500P and Ductibor500) during the forming process. Initially, specimens with varying notch sizes were designed and fabricated to perform uniaxial tensile tests to determine their mechanical properties. Then, the deformation process of the notched specimens was simulated using finite element software, revealing the distribution and variation of stress triaxiality at the fracture surface. By combining both experimental and simulation data, the parameters of the Johnson–Cook (J–C) damage model were calibrated, and the effects of temperature, strain rate, and stress triaxiality on material fracture behavior were further analyzed. Based on finite element analysis, the relevant coefficients for stress triaxiality, strain rate, and temperature were systematically calibrated, successfully establishing a J–C fracture criterion for TWB welds, Usibor1500P, and Ductibor500 high-strength steels. Finally, the calibrated damage model was further validated through the Nakajima-type bulge test, and the simulated Forming Limit Diagram (FLD) closely matched the experimental data. The results show that the analysis based on the J–C damage model can effectively predict the fracture behavior of tailor welded blanks (TWB) during the forming process. This study provides reliable numerical predictions for the damage behavior of high-strength steel laser-customized welded sheets and offers a theoretical basis for engineering design and material performance optimization.

## 1. Introduction

By using laser tailor welding, high-strength steel blanks of varying alloy compositions and strength grades can be fused to create a TWB structure with gradient properties [1,2,3]. High-strength steel TWBs, known for their outstanding mechanical properties, lightweight design, high-precision welding capabilities, and resistance to heat and corrosion [4,5], are increasingly being applied in industries such as automotive manufacturing and rail transportation. However, the material properties, especially the mechanical behavior and damage mechanisms under varying conditions, still require in-depth investigation to optimize the performance and reliability of TWBs in extreme environments [6,7,8].

The study of the forming performance of high-strength steel TWBs in the stamping process using experimental and numerical simulation methods has gained increasing attention. These approaches are capable of effectively assessing the formability of high-strength steel laser TWBs during the stamping process. Tang et al. [9]. investigated the hot stamping process of tailor welded high-strength steels, focusing on modeling and simulation of thermal, mechanical, and metallurgical behaviors, and evaluated the mechanical properties of the stamped automotive component, demonstrating the reliability of the simulation methods. Peister et al. [10] showed that TWBs produced from Ductibor500 and Usibor1500P have stable forming characteristics. They assessed how the thickness of the material affects the hardness of the parts and compared the results with those obtained using tailored in-die heating (IDH) techniques. Kinsey et al. [11] introduced a novel laser-tailored welded blank forming technique designed to enhance the accuracy and performance of the formed parts through innovative processes. Scott et al. [12] developed a numerical model of the weld seam line using finite element software LS-DYNA, enabling more accurate prediction of the performance of laser-tailored welded blanks during the forming process, thus improving the precision of numerical simulations. Tian et al. [13] examined the forming behavior of high-strength steel TWBs by utilizing a bulging test system. Zhang [14] introduced an optimization approach for TWB forming based on dynamic explicit finite element simulations, improving the efficiency of the optimization process and validating its effectiveness through stamping case studies. Chiu et al. [15] used both numerical simulations and experimental techniques to investigate the mechanical behavior and microstructural changes of TWBs throughout the hot stamping process. The research results indicate that the tailor welded blanks exhibit good formability and low crack sensitivity during hot stamping, providing a theoretical basis for the design of automotive structural components. Wang et al. [16] proposed an innovative method for controlling TWBs during the stamping and forming process, where they optimized the forming parameters using orthogonal and multi-objective tests, and confirmed the findings with experimental results.

The stamping and forming process of TWBs unavoidably causes material damage, which is mainly observed as the formation of micro-defects, including pores and cracks. These defects can further propagate under external forces, eventually leading to material fracture or failure. To address this, researchers have developed a macroscopic damage mechanics framework based on continuum mechanics, introducing a series of macroscopic state variables to effectively characterize the material’s damage effects, without delving into the microscopic physical mechanisms of damage evolution. The goal is to ensure that the macroscopic mechanical behavior predicted by the damage variables aligns closely with the actual performance of the material [17]. Johnson and Cook [18] proposed a comprehensive damage model that integrates continuum mechanics with viscoplastic mechanics. It is primarily used to predict the fracture behavior of metal sheets under extreme dynamic loading conditions, effectively addressing complex mechanical issues such as large plastic deformation and strain-rate sensitivity.

The damage evolution of the J–C damage model [18] is a function of plastic strain as the damage parameter, as shown in the following formula.

(1) $$D=\sum \frac{\Delta \varepsilon_{eq}}{\varepsilon_{f}}$$

where D represents the damage variable, $\Delta \varepsilon_{eq}$ is the increment of equivalent plastic strain (PEEQ), $\varepsilon_{f}$ is the equivalent fracture strain at the current cycle step. The damage variable starts at *D* = 0 and when *D* = 1, material failure occurs.

The equivalent plastic strain $\varepsilon_{\mathrm{eq}}$ [19,20] is calculated as follows:

(2) $$\varepsilon_{\mathrm{eq}}=[D_{1}+D_{2}\exp(D_{3}\eta )](1+D_{4}\ln{\dot{\varepsilon }}^{*})(1+D_{5}T^{*})$$

where $\varepsilon_{\mathrm{eq}}$ is PEEQ, *D*_1_ and *D*_2_ are constants related to the material properties, *D*_3_ is stress triaxiality sensitivity factor, *D*_4_ is strain rate sensitivity factor, *D*_5_ is temperature response coefficient, $\eta =\sigma_{m}/\sigma_{eq}$ represents stress triaxiality, $\sigma_{m}$ is mean principal stress, $\sigma_{eq}$ is Mises equivalent stress, ${\dot{\varepsilon }}^{*}=\dot{\varepsilon }/{\dot{\varepsilon }}_{0}$ represents dimensionless equivalent plastic strain rate, ${\dot{\varepsilon }}_{0}$ represents reference plastic strain rate; $\dot{\varepsilon }$ is current equivalent plastic strain rate; $T^{*}=(T-T_{r})/T_{m}-T_{r}$ represents the dimensionless temperature parameter, T is the test setting temperature; T_r_ is the reference temperature, taken as *T_r_* = 20 °C; Tm is the material’s melting point temperature (typically 1560 °C).

Researchers have widely used the Johnson–Cook (J–C) damage model for modeling and parameter calibration of the damage behavior of metal materials under complex loads. Through studies on different materials and experimental methods, the applicability and accuracy of this model in describing material fracture mechanisms have been validated. Hu et al. [19] conducted a set of mechanical experiments on Q345C steel using a material testing machine and a split Hopkinson pressure bar experimental setup, including quasi-static and dynamic compression and tensile tests. They obtained the quasi-static stress–strain curves of Q345C steel over a wide temperature range and systematically characterized the material’s fracture failure behavior under complex stress paths. According to these experimental findings, the parameters for the J–C constitutive model and the damage–fracture model were calibrated. Pandya et al. [21] used the J–C damage model to investigate the damage behavior of high-strength steel under dynamic loading conditions. They focused on the influence of material parameters on damage evolution and validated the effectiveness of the obtained damage model through a series of experiments. Li et al. [22] used the J–C damage model to analyze the failure mechanism of aluminum alloy under impact loading. The findings indicated that the strain rate and temperature conditions of the loaded material significantly influence the material’s damage mode. Based on this conclusion, they proposed corresponding suggestions for optimizing the forming process. Skripnyak [23] used the J–C damage model to analyze the mechanical properties of titanium alloy at high strain rates. Not only did they establish the corresponding fracture criterion, but they also validated the accuracy of the obtained damage model parameters through comparison with numerical simulations and experimental results. At the same time, Wang et al. [24] explored the application of the J–C damage model under complex loading conditions. Through experimental analysis of different metallic materials, they verified the model’s effectiveness in predicting material failure. Dhanraj et al. [25] explored the calibration issue of the Johnson–Cook damage model in scratch wear numerical simulations. They found that during the scratching process, the material’s stress state corresponds to a negative stress triaxiality, whereas traditional damage model calibration relies on positive values. To address this, they developed a new “scratch-based” calibration procedure and compared the crack trajectories obtained using different calibration methods. Bal et al. [26] systematically calibrated the J–C material constitutive parameters and damage model parameters (*D*_1_–*D*_5_) required for finite element simulation of Al 7068-T651 alloy through tensile tests under medium to high strain rates and high-temperature conditions. Optical microscopy was used to measure the fracture region, and combined with the Levenberg–Marquardt optimization method, factors such as stress triaxiality, rolling direction, strain rate, and temperature were considered. A J–C damage model suitable for high-precision simulations was established, providing a reliable basis for numerical predictions of this alloy in defense engineering.

TWBs are increasingly used due to their excellent performance. Their forming properties and damage mechanisms show significant differences compared to traditional homogeneous plates, especially in the weld seam and material transition zones, where stress concentrations and early failure are more likely to occur. The combination of different materials and thicknesses makes the mechanical response of TWBs more diverse under complex forming conditions, which raises higher demands on forming limits, fracture behavior, and damage evolution. Therefore, in-depth research on the constitutive relationships and damage models of TWBs, as well as the development of numerical prediction methods, has become a key focus for researchers. Zadpoor [27] discussed the numerical modeling work of TWBs, focusing on the finite element modeling of the welding area, the material models used in TWB numerical modeling, and key issues related to theoretical failure criteria. The study also explored the application, design, and optimization of the models in TWBs. Rajesh Kannan [28] examined the effect of the weld seam direction on the forming performance of AISI 316L welded sheets (TWBs). Through experiments and Johnson–Cook damage model finite element simulations, it was found that the weld seam direction significantly affects stress distribution and cup height prediction. The forming performance is more severely reduced when the weld seam is in the direction perpendicular to the rolling direction, with fracture occurring more easily on the thin sheet side. However, the fracture behavior of TWBs under different forming conditions has not been fully characterized, especially concerning factors such as stress triaxiality, strain rate, and temperature. This study aims to fill this gap by calibrating the J–C damage model and conducting a comprehensive analysis of TWB forming performance, focusing on the fracture mechanisms of Usibor1500P and Ductibor500 high-strength steel base materials and weld seams. The model is experimentally validated through a thermal Nakajima-type bulge test.

## 2. Uniaxial Tensile Experiments 

### 2.1. Experimental Design 

To accurately obtain the parameters of the J–C damage model, the experimental design needs to be carried out step by step to systematically calibrate the key parameters of the damage model that are influenced by factors such as stress triaxiality, temperature, and strain rate. Based on the current research by some scholars on the J–C damage model [29,30], the research design for this study is as follows: First, tensile tests are performed on notched specimens with different notch radii to determine the fracture strain under different stress triaxialities and further evaluate the J–C damage model parameters *D*_1_, *D*_2_ and *D*_3_. Then, tensile tests on smooth specimens are conducted at room temperature and strain rates of 0.01 s^−1^, 0.05 s^−1^ and 0.1 s^−1^ to calculate and determine the damage model parameter *D*_4_. Finally, at a constant strain rate of 0.001 s^−1^, tensile tests are conducted on smooth specimens at 600 °C, 700 °C, and 800 °C. Based on the experimental results, the damage model parameter *D*_5_ is calculated and determined. The specific experimental plan is shown in Table 1.

### 2.2. Experiment Specimens and Equipment 

**Figure 1:**
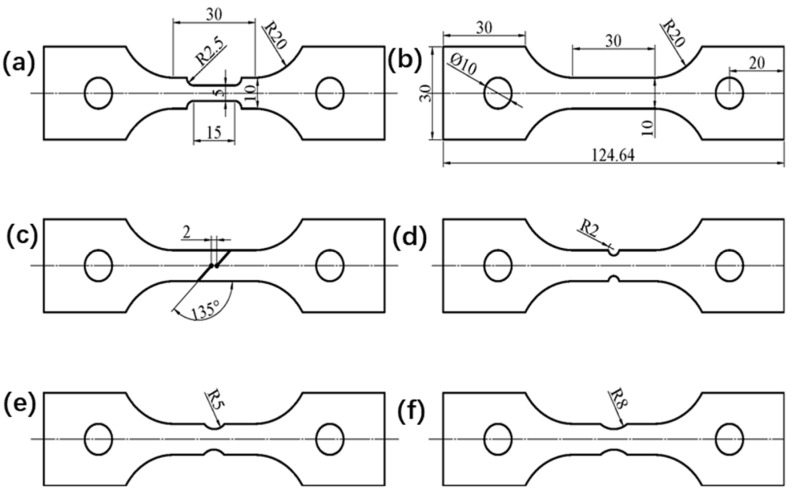
Geometry of specimens for tensile tests (unit: mm): (**a**) smooth specimens of the weld seam; (**b**) smooth specimens of base material; (**c**) shear specimen; (**d**) notched specimen with a notch radius of R2: (**e**) notched specimen with a notch radius of R5; (**f**) notched specimen with a notch radius of R8.

The tensile test was conducted on two base materials of TWBs: Usibor1500P and Ductibor500. These high-strength steel sheets, manufactured by ArcelorMittal S.A. (Luxembourg city), have a maximum tensile strength of 1500 MPa and 500 MPa at ambient temperature, respectively. Both materials feature a consistent thickness of 1.5 mm, with the test specimen’s length oriented along the rolling direction of the sheets. The chemical compositions of these sheets are detailed in Table 2 and Table 3. The dimensions and configuration of the hot tensile test specimens are illustrated in Figure 1.

The dimensions of the specimens required for the tensile tests are shown in Figure 1 according to the national standard GB/T 228-2002 [31] for tensile specimens. To comprehensively evaluate the material’s mechanical behavior under different stress state conditions, three types of tensile specimens were designed. Figure 1a,b show smooth specimens of the weld seam and base material, respectively; Figure 1c shows a shear specimen, where the fracture zone is aligned with the test load direction, designed to simulate the fracture characteristics of the material under shear stress. Figure 1d–f show notched specimens, with the fracture zone perpendicular to the test load direction. The purpose of the notch design is to introduce stress concentration in the specimen, thereby exploring the effect of the notch radius on the material’s fracture toughness and strength. The notch radii are set to 2 mm, 5 mm, and 8 mm, respectively.

The WDW-100E tensile tester (Jinan Hengda Huifeng Testing Instrument Co., Ltd., Jinan, China) is used for the tensile test to investigate the mechanical behavior of the two base materials and weld seam. Equipped with a temperature control system, the tensile testing equipment utilizes a thermocouple inside the heating hood to provide real-time temperature feedback to the display meter’s temperature control mechanism. Furthermore, the computer control system enables accurate adjustment of both displacement and deformation speed during the tensile test, aiding in the collection and analysis of the resulting data curve. 

### 2.3. Analysis of Experimental Results 

**Figure 2:**
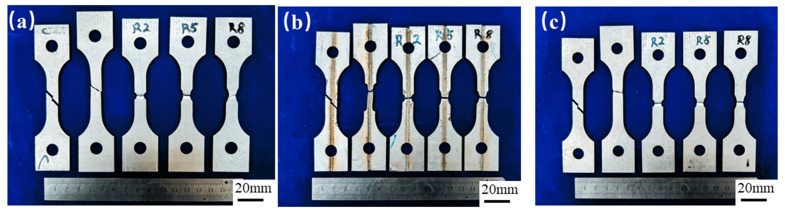
Fractured samples in tensile test ($\dot{\varepsilon }\;=\;0.001$ s^−1^): (**a**) Usibor1500P; (**b**) weld seam; (**c**) Dustibor500.

The experiment used uniaxial tensile testing, with quasi-static loading applied at a constant rate of 1.5 mm/min at room temperature (20 °C). For specimens with different geometric dimensions, each group was tested three times, and the arithmetic mean was taken to reduce the impact of random errors on the results. Figure 2 shows the fractured samples after tensile testing.

**Figure 3:**
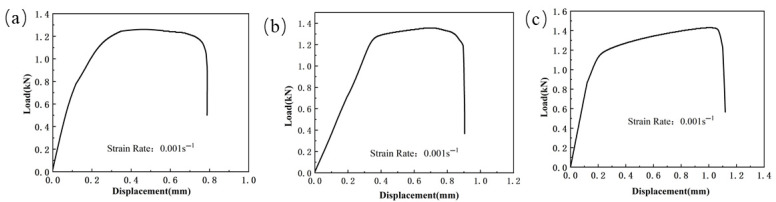
The load–displacement curve of the shear specimen tensile test for TWBs. ($\dot{\varepsilon }\;=\;0.001$ s^−1^): (**a**) Usibor1500P; (**b**) Weld seam; (**c**) Dustibor500.

**Figure 4:**
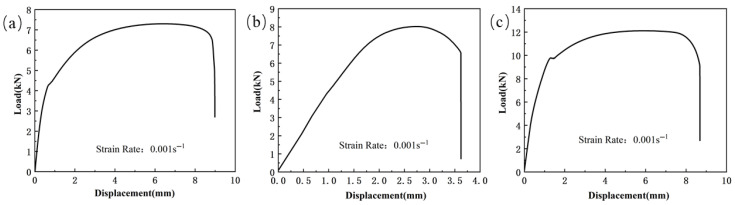
The load–displacement curve of the smooth specimen tensile test for TWBs ($\dot{\varepsilon }\;=\;0.001$ s^−1^): (**a**) Usibor1500P; (**b**) weld seam; (**c**) Dustibor500.

**Figure 5:**
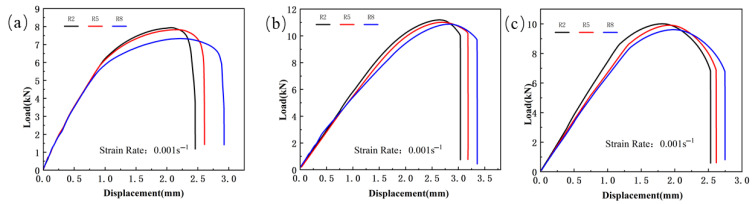
The load–displacement curve of the notched specimen tensile test for TWBs ($\dot{\varepsilon }\;=\;0.001$ s^−1^): (**a**) Usibor1500P; (**b**) weld seam; (**c**) Dustibor500.

The load vs. displacement curves for the specimens can be seen in Figure 3, Figure 4 and Figure 5. Due to the abrupt change in the slope of the curve at fracture, the fracture displacement points of the test specimens can be extracted. The load–displacement curve of the shear specimen tensile test is shown in Figure 3. From the Figure 3, it can be seen that the maximum shear strength of the weld seam, Usibor1500P and Ductibor500 does not differ significantly, indicating that these materials have similar performance in terms of shear strength. However, they exhibit different ductilities, with fracture displacements of 0.887 mm, 0.786 mm, and 1.024 mm, respectively.

The load–displacement curve of the smooth specimen tensile test is shown in Figure 4. From Figure 4b, it can be seen that the welded specimen does not experience a significant hardening phase during the tensile test, and the increase in load is relatively gradual. This suggests that the material has poor plastic deformation capacity, possibly due to low ductility and insufficient resistance to deformation, leading to failure at a relatively low displacement. As the load increases, the weld specimen suddenly fractures after reaching a certain stress level. In contrast, the two base materials (Usibor1500P and Ductibor500) undergo a complete elastic, plastic, and fracture stage during the tensile process. In the elastic stage, the base materials exhibit a good linear response, followed by the plastic deformation stage, where the material begins to undergo nonlinear deformation. During this stage, the internal structure of the material changes, with dislocation motion and lattice slip leading to material hardening. Finally, after reaching the critical stress, the base material fractures, indicating the material’s ultimate load-bearing capacity.

The load–displacement curve of the notched specimen tensile test is shown in Figure 5. From the curve, it can be seen that as the notch radius increases, the tensile strength of all three materials shows a decreasing trend. Specifically, the peak load of Usibor1500P high-strength steel decreases from 7.94 kN to 7.32 kN, the peak load of Ductibor500 high-strength steel decreases from 10.01 kN to 9.6 kN, and the peak load of the weld material decreases from 11.12 kN to 10.81 kN. Meanwhile, the fracture displacement shows a significant increase. This phenomenon is mainly due to the presence of the notch, which alters the internal stress distribution of the material, especially at the root of the notch. As the notch radius increases, the degree of stress concentration decreases, leading to material yielding at lower stress levels, thus reducing the tensile strength of the material. At the same time, a larger notch radius also implies a larger plastic deformation region, which may lead to an increase in fracture displacement, thereby affecting the material’s fracture behavior.

### 2.4. Analysis of the Stress Triaxiality 

**Figure 6:**
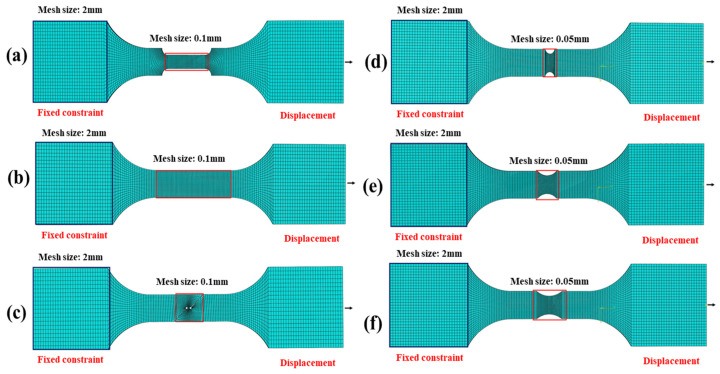
The mesh divisions of various tensile specimens: (**a**) welded smooth specimen; (**b**) base material smooth specimen; (**c**) shear specimen; (**d**) R2 notched specimens; (**e**) R5 notched specimen; (**f**) R8 notched specimen.

The static tensile test was numerically simulated using the commercial finite element software ABAQUS 6.14 to obtain fracture strain and stress triaxiality values under different stress states. In Abaqus/Explicit 6.14, the deformation process was simulated. To balance calculation accuracy and efficiency, the element type used was an 8-node reduced integration solid element (C3D8R). During the tensile process, significant plastic deformation is expected in the middle region of the specimen, so a refined mesh was applied to this area. For the model boundary conditions, the fixture at the specimen clamping end was simulated with full-degree-of-freedom constraints to mimic the fixing constraints of the testing machine, ensuring the stability of the boundary conditions. The axial displacement load was applied to the other end using a reference point coupling method to achieve uniaxial tension. The loading time and displacement were set to match the experimental conditions, ensuring that the simulation results were highly comparable to the actual test data. Figure 6 shows the mesh divisions for various tensile specimens.

**Figure 7:**
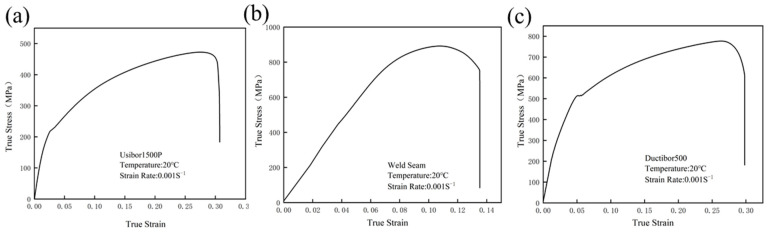
True stress–strain curve during the tensile test of the smooth specimen (20 °C, $\dot{\varepsilon }\;=\;0.001$ s^−1^): (**a**) Usibor1500P; (**b**) weld seam; (**c**) Dustibor500.

**Figure 8:**
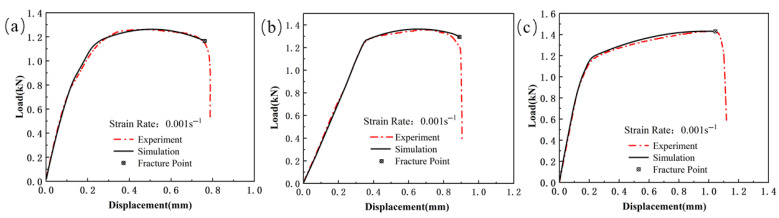
Comparison of the load–displacement curves between the simulation and the experiment for the shear specimen. ($\dot{\varepsilon }\;=\;0.001$ s^−1^): (**a**) Usibor1500P; (**b**) weld seam; (**c**) Dustibor500.

**Figure 9:**
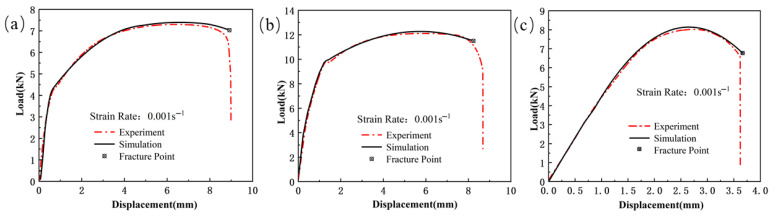
Comparison of the load–displacement curves between the simulation and the experiment for the smooth specimen. ($\dot{\varepsilon }\;=\;0.001$ s^−1^): (**a**) Usibor1500P; (**b**) weld seam; (**c**) Dustibor500.

**Figure 10:**
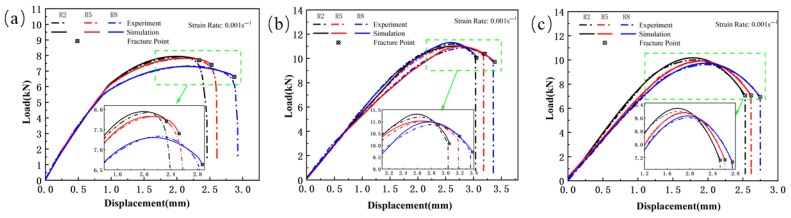
Comparison of the load–displacement curves between the simulation and the experiment for the notched specimen. ($\dot{\varepsilon }\;=\;0.001$ s^−1^): (**a**) Usibor1500P; (**b**) weld seam; (**c**) Dustibor500.

Tensile tests were conducted on the smooth specimen at room temperature with a strain rate of 0.001 s^−1^, and the measured true stress–strain data were used as the material constitutive model input parameter, as shown in Figure 7. Then, its tensile deformation is simulated, and the load–displacement curve of the specimen is extracted and compared with the actual experimental results for analysis. In the simulation of the shear specimen, there is a significant error in the comparison of the displacement-load curve, with the simulated curve showing higher values. This is due to the stress state dependence of the material constitutive relationship [32,33]. To address this issue, the stress is reduced before using the stress–strain curve of the smooth specimen as the material parameter for the finite element model. Multiple trial calculations show that the real stress values obtained from the smooth specimen should be multiplied by a correction factor of 0.86 before being input into the finite element system as material property parameters. The consistency between the numerical calculation results of the shear specimen and the experimental data has significantly improved. The final results are shown in Figure 8, Figure 9 and Figure 10, where the load–displacement curves obtained from the numerical simulation match the experimental responses well.

**Figure 11:**
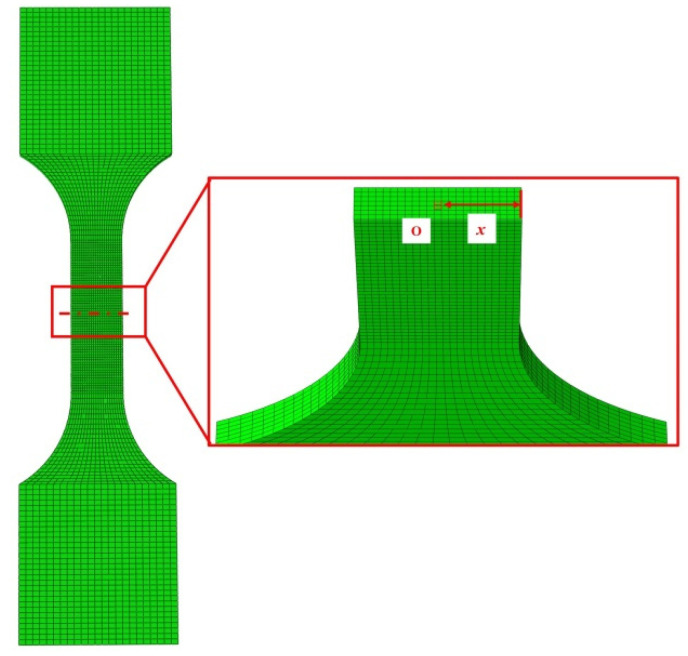
Schematic diagram of the specimen’s minimum cross-section.

**Figure 12:**
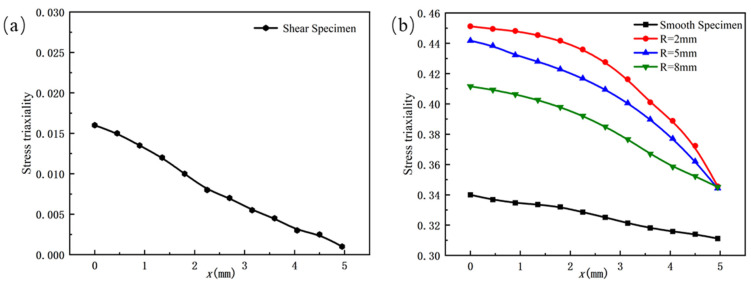
The distribution of stress triaxiality at the minimum cross-section for each type of specimen: (**a**) shear specimen; (**b**) smooth specimen and notched specimens.

Based on the analysis of the load–displacement curve obtained from the experiment, it can be seen that when the specimen fractures, the slope of the curve exhibits a significant abrupt change. This moment corresponds to the point where the stress triaxiality and fracture strain values are obtained. The stress triaxiality extraction position is selected using the minimum cross-section method. The principle is to capture the evolution of the stress state in the minimum cross-sectional area of the specimen during the tensile process through finite element simulation. As shown in Figure 11, the definition of x is the radial distance from the center position to each point on the cross-section. As shown in Figure 12, the stress triaxiality exhibits a typical non-uniform distribution along the cross-section. The numerical simulation results indicate that the stress triaxiality reaches its maximum value at the center, and the value decreases as it approaches the edges. Under the tensile load, the central region of the material experiences multi-axial constraint, causing it to preferentially enter a triaxial tensile stress state, thus becoming the initiation point for crack nucleation and propagation. Therefore, the center of the specimen’s minimum cross-section is selected as the data extraction point. In addition, comparative analysis reveals significant differences in the fracture characteristics between the conventional tensile specimen and the shear specimen. The former exhibits cross-sectional contraction during necking, with a noticeable variation in stress triaxiality. On the other hand, the shear specimen, constrained by a smaller fracture cross-sectional size, effectively limits the local development of strain, causing the stress triaxiality to remain relatively stable within a smaller range. As a result, the distribution of stress triaxiality on the shear fracture cross-section is relatively uniform.

**Figure 13:**
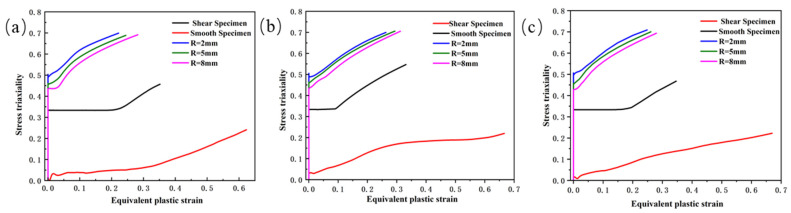
The relationship curve between stress triaxiality and PEEQ for each type of specimen ($\dot{\varepsilon }\;=\;0.001$ s^−1^): (**a**) Usibor1500P; (**b**) weld seam; (**c**) Dustibor500.

Figure 13 shows the curve of stress triaxiality versus PEEQ for the high-strength steel laser-welded plate. The smaller the notch radius of the sample, the relatively higher the corresponding stress triaxiality. Throughout the entire process from the start of tensile loading to fracture, the stress triaxiality at the fracture site of each specimen continuously changes dynamically. To accurately quantify the specific value of this stress triaxiality, the average stress triaxiality is used for the relevant calculations, and its mathematical expression [34] is defined as:

(3) $$\eta_{w}=\frac{1}{\varepsilon_{f}}\int_{0}^{\varepsilon_{f}}\frac{\sigma_{m}}{\sigma_{eq}}d\varepsilon_{p}$$

where $\varepsilon_{f}$ represents the fracture strain, $\sigma_{m}$ represents mean principal stress, and $\sigma_{eq}$ represents the Mises equivalent stress.

After determining the stress triaxiality, the fracture strain of the material must be determined simultaneously. Based on the characteristics of the experimental data, the displacement corresponding to the abrupt change point in the slope of the load–displacement curve obtained from the experiment is defined as the fracture initiation displacement. The PEEQ value at the moment of fracture in the corresponding experiment is then extracted to obtain the fracture strain value of the specimen.

**Figure 14:**
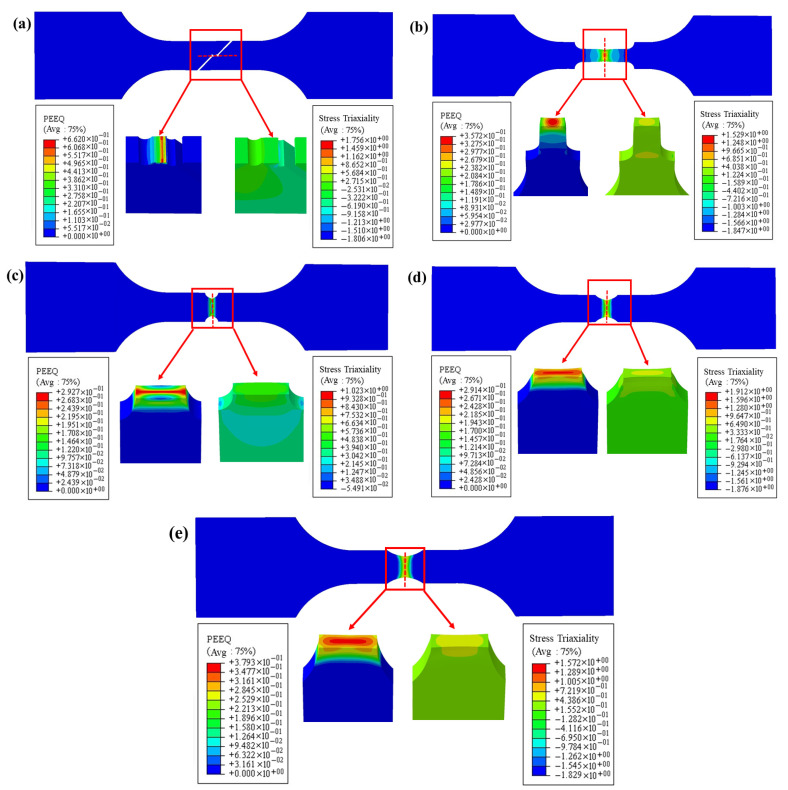
Distribution of PEEQ and stress triaxiality at the moment of specimen fracture: (**a**) shear; (**b**) smooth; (**c**) R2 notch; (**d**) R5 notch; (**e**) R8 notch.

Since the stress triaxiality variations of the two base materials and the weld tend to be consistent, the PEEQ and stress triaxiality distribution contour maps at the moment of specimen fracture failure were extracted using the weld as an example, as shown in Figure 14. As seen in Figure 14a, the distribution of PEEQ on the fracture surface of the shear specimen shows significant variability, making it challenging to extract the PEEQ points from the fracture zone. Therefore, the PEEQ on the cross-section of the fracture zone was averaged and used as the fracture strain for the shear specimen, resulting in an average PEEQ of 0.663. Additionally, since the stress triaxiality is relatively evenly distributed across the fracture surface, the stress triaxiality value at the center of the fracture surface can be used to represent the overall stress triaxiality level of the shear specimen. By calculating with the average stress triaxiality, the result obtained is 0.051.

As seen in Figure 14b, on the fracture surface of the smooth specimen, the distribution of PEEQ and stress triaxiality is mainly concentrated in the geometric center region. In this area, due to the relatively high stress triaxiality, the plastic deformation of the material becomes more pronounced, leading to an increase in the PEEQ value. This distribution characteristic indicates that during the tensile process, the central region of the specimen experienced a larger multiaxial stress state, which promoted the localization of plastic deformation. As seen in Figure 14c–e, the variation in notch radius directly affects the stress triaxiality, which in turn influences the material’s plastic deformation and fracture characteristics. A smaller notch radius is associated with higher stress concentration, leading to higher stress triaxiality and earlier fracture occurrence, causing the specimen to neck earlier. In contrast, a larger notch radius results in lower stress triaxiality, reducing the level of stress concentration and allowing the material to withstand more plastic deformation before fracture. Table 4 lists the average stress triaxiality and fracture strain data obtained from simulations for different types of specimens.

## 3. Fracture Prediction by Uniaxial Tensile Test of TWBs 

### 3.1. Determination of J–C Damage Model Parameters

(1) Determination of *D*_1_, *D*_2_ and *D*_3_

In the process of identifying the stress triaxiality factor using the J–C damage model, the temperature and strain rate factors are neglected. Therefore, Equation (2) can be simplified as:

(4) $$\varepsilon_{\mathrm{eq}}=[D_{1}+D_{2}\exp(D_{3}\eta )]$$

**Figure 15:**
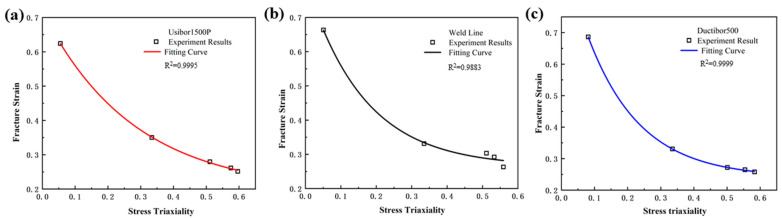
The Fitting Relationship Curve between Stress Triaxiality and Fracture Strain: (**a**) Usibor1500P; (**b**) Weld seam; (**c**) Dustibor500.

The stress triaxiality and fracture strain parameters of each specimen obtained from the simulation in Table 4 are used as feature variables to fit the equation. The fitting results are shown in Figure 15, and the parameters *D*_1_, *D*_2_ and *D*_3_ for TWBs obtained from the fitting are shown in Table 5.

As shown in Figure 15, under the same strain rate conditions, the fracture strain of the material decreases as the stress triaxiality increases. This indicates that the stress state of the material has a significant impact on its fracture failure strain. From the observed decreasing trend, it can be seen that when the stress triaxiality is in the range of 0 to 0.5, the fracture strain decreases relatively quickly, while in the range of 0.5 to 0.6, the change in fracture strain becomes more gradual. This is because, when high-strength steel materials are subjected to external loading, internal stresses are generated within the material. Stress triaxiality refers to the differences in the magnitude and direction of stress acting on the material in different directions. When the stress triaxiality is high, the internal stress distribution of the material becomes uneven, leading to stress concentration, which in turn accelerates fatigue cracking and fracture failure. This uneven stress distribution causes micro-defects in the material to more easily propagate and accumulate, thereby reducing the material’s fracture strain.

(2) Determination of *D*_4_

Based on the determination of *D*_1_, *D*_2_, and *D*_3_, the effect of temperature on the material’s fracture behavior is neglected, and the strain rate factor *D*_4_ is calibrated. The J–C damage model expression can be simplified as:

(5) $$\varepsilon_{\mathrm{eq}}=[D_{1}+D_{2}\exp(D_{3}\eta )](1+D_{4}\ln{\dot{\varepsilon }}^{*})$$

**Figure 16:**
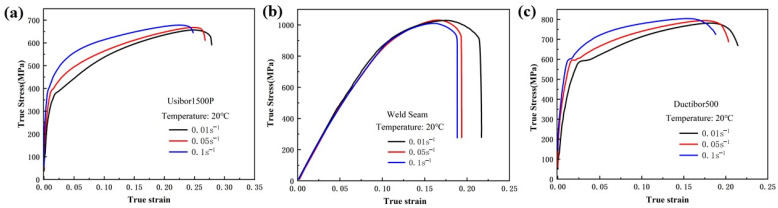
Tensile test curves of smooth samples under different strain rates at room temperature: (**a**) Usibor1500P; (**b**) Weld seam; (**c**) Dustibor500.

Tensile tests were conducted at room temperature and strain rates of 0.01 s^−1^, 0.05 s^−1^, and 0.1 s^−1^, with a reference strain rate of 0.001 s^−1^, to obtain the true stress–strain curves under different strain rate conditions. The results are shown in Figure 16. The fracture strain values for the smooth samples at different strain rates are shown in Table 6.

**Figure 17:**
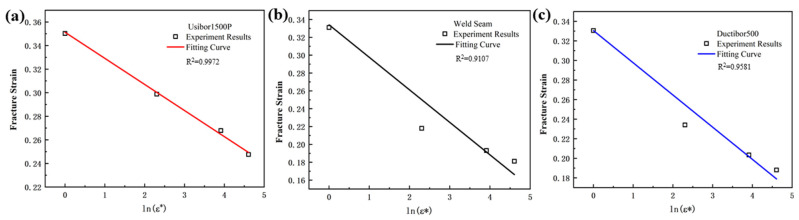
Strain rate and fracture strain relationship curve: (**a**) Usibor1500P; (**b**) Weld seam; (**c**) Dustibor500.

The stress triaxiality and fracture strain parameters of the smooth samples under different strain rate conditions were substituted into Equation (5) for fitting. The fitting results are shown in Figure 17, and the parameter *D*_4_ for TWBs obtained from the fitting is shown in Table 7.

Figure 17 shows that as the strain rate decreases, the fracture strain increases. This is due to the fact that at slower deformation rates, the material has more time to undergo plastic deformation, which helps to distribute the stress and minimize crack propagation. This enables the material to withstand larger deformations before fracturing. Conversely, at higher strain rates, the fracture strain is typically smaller, as rapid loading promotes brittle fracture, limiting the material’s ability to undergo effective plastic deformation.

(3) Determination of *D*_5_

When calibrating temperature factors, if the influence of strain rate on the material’s fracture behavior is neglected, the J–C fracture criterion can be simplified as follows:

(6) $$\varepsilon_{\mathrm{eq}}=[D_{1}+D_{2}\exp(D_{3}\eta )](1+D_{5}T^{*})$$

**Figure 18:**
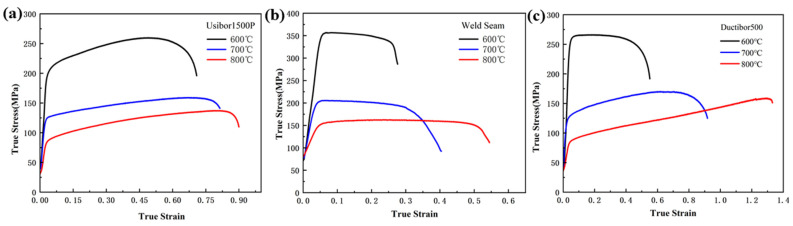
True stress–strain curves of smooth samples under static tensile conditions at different temperatures: (**a**) Usibor1500P; (**b**) weld seam; (**c**) Dustibor500.

Under static tensile conditions, uniaxial hot tensile tests were conducted on smooth samples at temperatures of 600 °C, 700 °C and 800 °C, with a reference temperature of 20 °C. The true stress–strain curves under different temperature conditions are shown in Figure 18. The fracture strain values for the smooth samples were extracted from the curves and are presented in Table 8.

**Figure 19:**
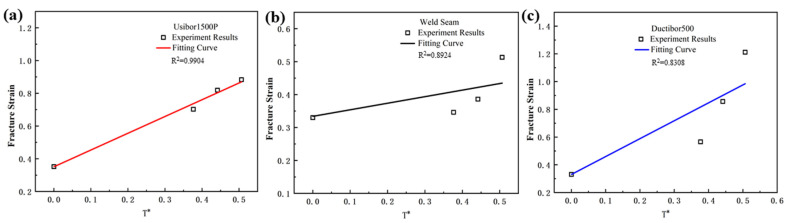
The relationship curve between temperature and fracture strain: (**a**) Usibor1500P; (**b**) weld seam; (**c**) Dustibor500.

The stress triaxiality and fracture strain parameters of the smooth samples under different temperature conditions were substituted into Equation (6) for fitting. The fitting results are shown in Figure 19, and the parameter *D*_5_ for TWBs obtained from the fitting is shown in Table 9.

Figure 19 shows that temperature has a significant impact on the fracture strain of high-strength steel materials. As the temperature increases, the fracture strain of the material tends to increase. This is due to the enhanced thermal vibration of atoms and grains at higher temperatures, which improves the material’s ability to undergo plastic deformation. Additionally, the internal stresses within the material decrease at high temperatures, effectively suppressing the nucleation and propagation of microcracks, thereby reducing the material’s brittleness. As a result, the material becomes more prone to plastic deformation during loading and is less likely to fracture.

The five parameters of the J–C damage model for TWBs are shown in Table 9. It can be seen that the *D*_5_ parameters of the two base materials are significantly greater than those of the weld, indicating that the base material is more sensitive to temperature, leading to a more pronounced softening effect at high temperatures and a larger fracture strain.

### 3.2. Tensile Specimen Fracture Analysis

In order to illustrate the evolution of the microstructure under different deformation conditions, the relationship between the macro phenomena of high-strength steel forming and the micro changes is analyzed. The fracture morphology of the smooth specimen butt-welded plate under a strain rate of 0.1 s^−1^ was analyzed. 

**Figure 20:**
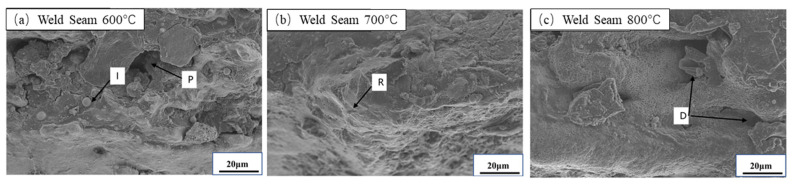
SEM fracture topography weld seam×1000 ($\dot{\varepsilon }\;=\;0.1$ s^−1^). Following features are marked: D—Dimple, R—River pattern, I—Inclusion, P—Porosity defect.

As shown in Figure 20a, at 600 °C, the fracture surface exhibits prominent porosity defects and inclusions. This indicates that during tensile deformation, due to weak interfacial bonding strength, inclusions tend to debond or fracture from the matrix, resulting in the formation of micro-voids. These voids gradually coalesce under loading and eventually evolve into main cracks that lead to fracture. The overall fracture morphology is predominantly brittle, suggesting that the material at this temperature lacks sufficient plasticity and is prone to crack initiation at defect locations.

When the temperature increases to 700 °C, as shown in Figure 20b, the fracture surface displays typical “river patterns”, which are characteristic of quasi-cleavage fracture. At this stage, some localized plastic deformation accompanies crack propagation, but the dominant mechanism remains crack extension along slip planes within grains. This indicates that the material possesses some degree of plasticity at 700 °C, though the overall fracture behavior still reflects moderate toughness.

Under high-temperature conditions of 800 °C, as shown in Figure 20c, the fracture surface presents a large number of uniformly distributed dimples, which is a typical morphology of ductile fracture. Elevated temperatures promote dislocation motion, making it easier for micro-voids to nucleate, grow, and coalesce, thereby enhancing the ductility of the material. This mechanism shows that the material under high temperature has excellent plastic deformation ability, allowing it to absorb more deformation energy and delay crack instability and propagation.

Based on the above analysis, it is clear that as the temperature increases from 600 °C to 800 °C, the fracture mechanism of the weld metal gradually transitions from brittle fracture to quasi-cleavage fracture, and eventually to dimple-dominated ductile fracture. The increase in temperature significantly improves the material’s plastic deformation capacity and fracture toughness.

### 3.3. Verification of the J–C Damage Model 

The damage model for TWBs is established based on uniaxial tensile test data, with the damage parameters determined through finite element simulations of uniaxial tension. Before extending the application of this model to predict fracture behavior in complex sheet forming processes, it is essential to evaluate its predictive reliability under representative stress triaxiality conditions. Therefore, TWB specimens subjected to hot tensile loading at 700 °C were selected as the study objects, and the J–C damage model was systematically validated under different stress states during uniaxial tensile tests.

**Figure 21:**
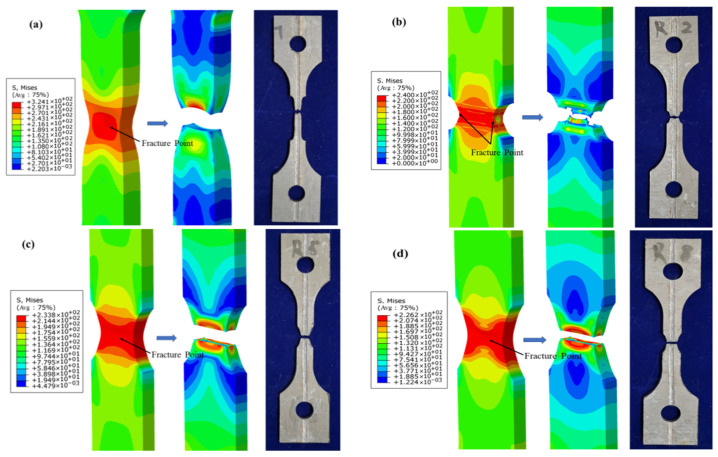
Comparison between the numerical simulation and experimental results of tensile testing for welded specimens: (**a**) smooth specimen; (**b**) R2 notched specimen; (**c**) R5 notched specimen; (**d**) R8 notched specimen.

**Figure 22:**
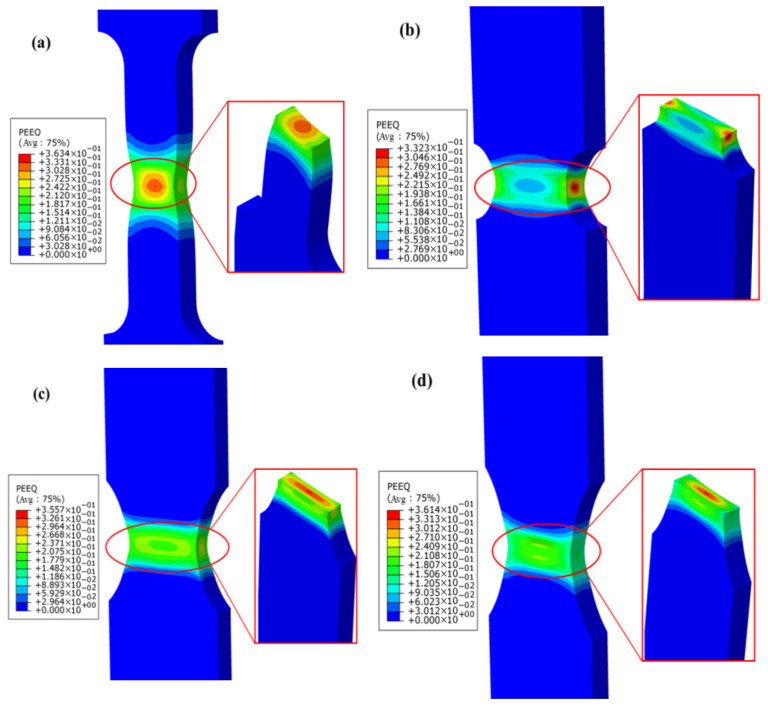
Simulation results of the PEEQ at the fracture initiation point of tensile testing for welded specimens: (**a**) smooth specimen; (**b**) R2 notched specimen; (**c**) R5 notched specimen; (**d**) R8 notched specimen.

The numerical simulation of the tensile test for the welded specimens was performed by importing the J–C model parameters, with the simulation results shown in Figure 21 and Figure 22. The results reveal the distribution characteristics of Mises stress and PEEQ on the fracture surfaces of the smooth specimens. Notably, significant stress concentration phenomena appeared in the central region of the geometry for the smooth, R5, and R8 specimens. This finding indicates that the material’s ductile fracture tends to initiate from the internal center of the smallest cross-sectional area, and the crack propagation path is perpendicular to the applied tensile direction, ultimately leading to macro-scale fracture of the specimen. In the R2 specimen, however, the PEEQ concentrated at both ends of the notch. The crack initiated from both sides, near the surface of the smallest cross-sectional area, and propagated toward the center of the specimen. Moreover, the simulation results for the notched specimens also demonstrated that the fracture occurred at the smallest cross-sectional area of the notch. These simulation results are in good agreement with the fracture failure observed in the experimental tests.

**Figure 23:**
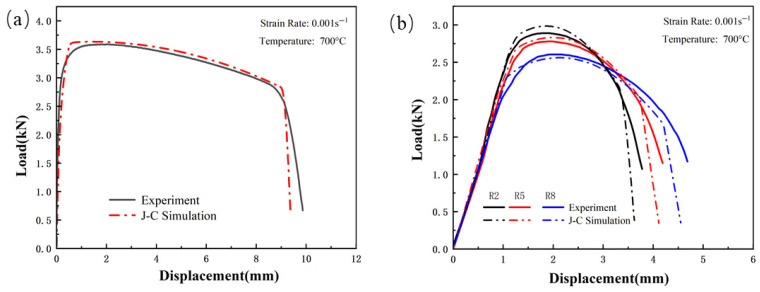
Comparison of Load–Displacement Curves Between High-Strength Steel Usibor1500P Tensile Test and J–C Simulation; (**a**) Smooth specimen; (**b**) Notched Specimens.

**Figure 24:**
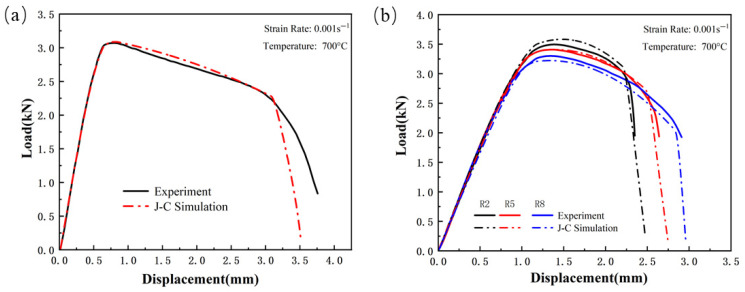
Comparison of Load–Displacement Curves Between Weld Seam Tensile Test and J–C Simulation: (**a**) Smooth specimen; (**b**) Notched Specimens.

**Figure 25:**
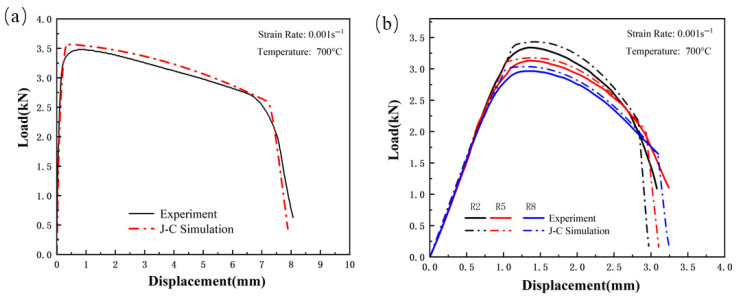
Comparison of Load–Displacement Curves Between High-Strength Steel Ductibor500 Tensile Test and J–C Simulation; (**a**) Smooth specimen; (**b**) Notched Specimens.

Figure 23, Figure 24 and Figure 25 show the load–displacement curves for TWBs under tensile loading, as predicted by the finite element simulation. It can be observed that the simulated load–displacement curves closely match the experimental curves. The combination of the specimen’s geometry and the direction of load application results in the material experiencing tensile stress under relatively high triaxial stress conditions, with tensile stress being the dominant state. Under these tensile stress conditions, although both the experimental and simulated curves exhibit a similar overall trend, slight differences are still present.

**Figure 26:**
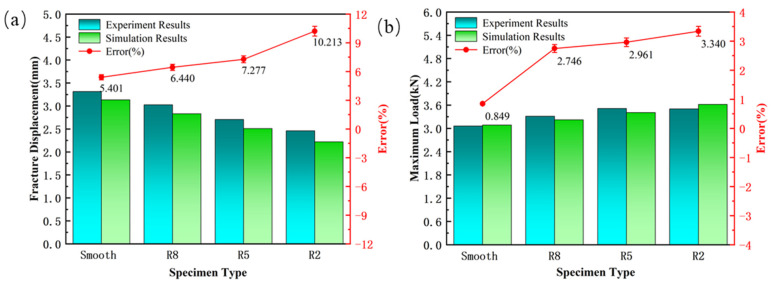
Comparison of J–C Model Predicted Fracture Displacement and Maximum Load with Experimental Results and Corresponding Error Curves: (**a**) Fracture Displacement; (**b**) Maximum Load.

Taking the welded seam as an example, Figure 26 analyzes key characteristic parameters during the tensile process, including fracture displacement and the maximum load-bearing capacity. The results show that the R2 notch sample has the largest prediction error, with fracture displacement and maximum bearing capacity prediction errors of 10.213% and 3.34%, respectively; in contrast, the smooth sample has the smallest prediction errors, 5.401% and 0.849%, respectively. These results indicate that the presence of the notch and its geometric size have a significant impact on the simulation prediction accuracy. From the image analysis, it can be seen that as the stress triaxiality increases, the prediction error shows an upward trend: the fracture displacement prediction error increases from 5.401% to 10.213%, and the maximum load prediction error increases from 0.849% to 3.34%. This trend is reflected in the simulation analysis of both Usibor1500P and Ductibor500 high-strength steels. This error is mainly due to the stress concentration and geometric size changes caused by the notch, leading to an uneven local stress distribution. Meanwhile, as the stress triaxiality increases, the material’s fracture behavior shifts from ductile fracture to brittle fracture, which in turn affects the accuracy of the simulation prediction.

**Figure 27:**
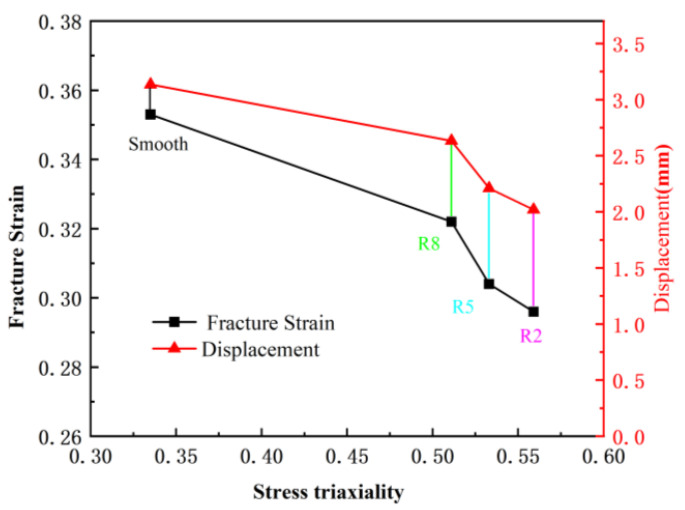
Fracture Strain and Displacement vs. Stress Triaxiality Relationship Curve at Final Fracture of the First Failure Element.

Figure 27 shows the relationship curve of fracture strain, displacement, and stress triaxiality at the final fracture of the first failure element in the tensile test of the welded specimen. The analysis results indicate that as the notch radius of the specimen decreases, both the fracture strain and displacement at final fracture decrease. Meanwhile, the stress triaxiality increases. This suggests that the level of stress triaxiality has a significant impact on the material’s fracture behavior. Under high stress triaxiality, both the material’s plastic deformation capability and ductility are suppressed, making the material more prone to brittle fracture. In contrast, under low stress triaxiality, ductile fracture is more likely to occur. Therefore, by appropriately adjusting the notch radius, it is possible to effectively control the stress triaxiality within the material, and thus, to some extent, regulate the material’s fracture mode.

The fracture failure criterion of high-strength steel TWBs under uniaxial tensile conditions was verified through simulation and experiments. The J–C damage model can accurately predict the fracture location and load–displacement curves for both smooth and notched specimens.

## 4. High-Temperature Nakajima-Type Bulging Experiments and Numerical Model Setup for TWBs

The earlier discussion concentrated on how the J–C damage parameters affect the simulation outcomes of TWBs under uniaxial tensile conditions. The size of the notch, the level of stress triaxiality, and temperature all have a significant impact on the accuracy of the predictions. In order to gain a deeper understanding of the damage mechanism in TWBs, this study utilized the J–C damage model and performed both hot Nakajima-type bulging tests and numerical simulations to thoroughly examine the progression of TWB damage under varying tensile conditions.

### 4.1. Materials and Experimental Platforms 

**Figure 28:**
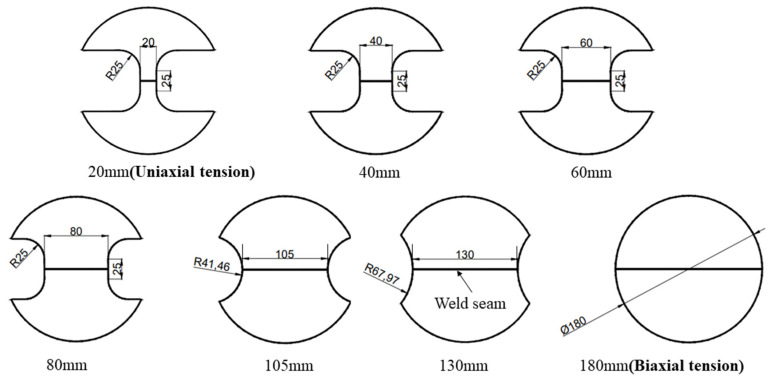
Geometry and measurements of the hot Nakajima-type bulging test specimen.

This test used TWBs made from Usibor1500P and Ductibor500 base materials, with their chemical compositions presented in Table 2 and Table 3. The design of the hot Nakajima-type bulging test specimens follows the reference standard (GB/T 24171.2-2009 [35]/ISO 12004-2:2008 [36]). The outer diameter dimensions are shown in Figure 28, where the design includes specimens with varying widths (20, 40, 60, 80, 105, 130 and 180 mm), each representing different strain paths. The increase in specimen width with the parallel section indicates the transition from uniaxial tension to biaxial tension [37].

The test was conducted using an EC600H sheet metal forming machine (Genbon Testing Equipment Co., Ltd., Changzhou, China). Prior to the forming limit test, a specific grid pattern was printed on the test specimen using the SX2-12–10A box resistance muffle furnace. The specimen was heated to 950 °C for 3 min, after which it was removed and allowed to cool to 700 °C. Once the specimen reached the required temperature, it was placed into the forming limit test machine. The test parameters included a stamping speed of 5 mm/s and a crushing force of 200 kN. At the conclusion of the test, the major and minor strains in the fracture zone were analyzed using the ARGUS v6.3.1-64bit strain analysis software, allowing the FLD curves for TWBs at 700 °C to be generated.

### 4.2. Numerical Parameters for Finite Element Analysis 

**Figure 29:**
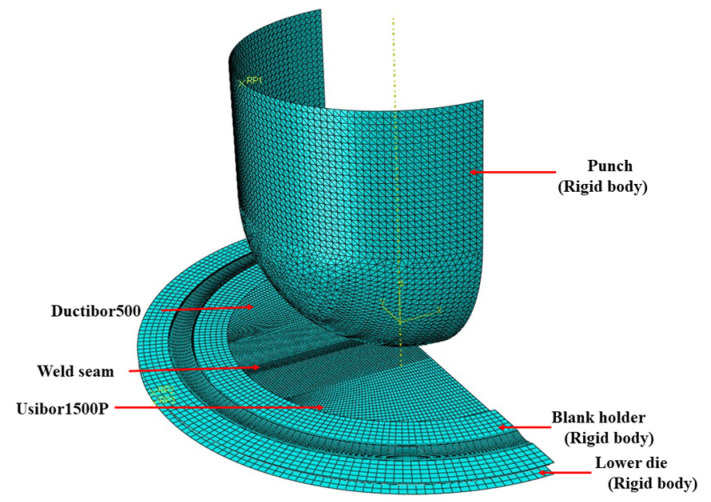
Finite element model for hot Nakajima-type bulging tests of TWBs.

In ABAQUS software, the entire stamping die is modeled as a discrete rigid body. For TWBs, it is modeled as a deformable shell. The weld settings are consistent with those in the uniaxial tensile simulation of the spliced plate. The expansion test simulation model uses material parameters based on the J–C damage model. S4RT shell elements are assigned to the mesh type of the base material on both sides of the TWBs. The ABAQUS/Explicit dynamic solver is then used for the calculation, as shown in Figure 29. Related thermophysical parameters are shown in Table 10.

## 5. Results and Discussions 

**Figure 30:**
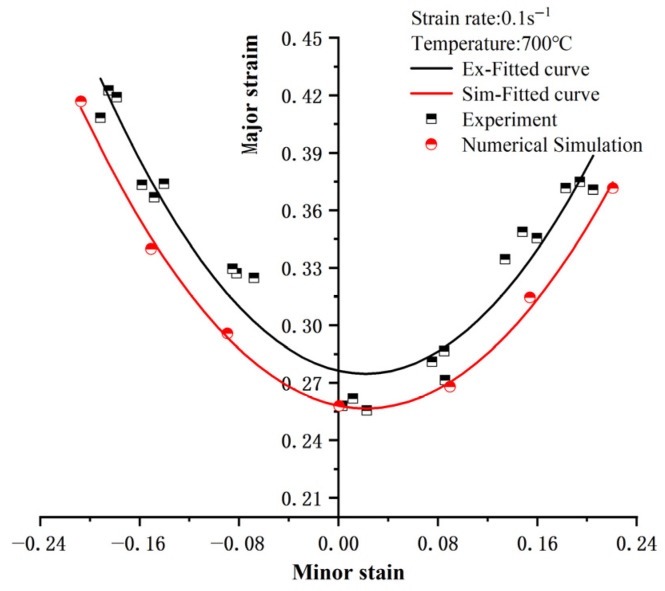
FLD of Experimental Data and Simulation Results.

The FLD (Forming Limit Diagram) is a graph used to describe the maximum plastic deformation a material can withstand during the forming process. It typically uses minor strain as the horizontal axis and major strain as the vertical axis. Major strain usually represents the degree of plastic deformation in the primary direction of the material during the forming process, while minor strain represents the degree of plastic deformation in the direction perpendicular to the primary deformation direction. The forming limits of the material are defined by different combinations of strain. The FLD helps describe the material’s plastic deformation ability in different directions, clarifying the forming limits of the material under specific strain combinations. Exceeding these limits results in rupture or failure. From Figure 30, it can be seen that the numerical simulation curve is in excellent agreement with the experimental data fitting curve. The trends of both curves match perfectly, demonstrating the accuracy of the numerical simulation based on the J–C damage model in predicting the forming limits of materials. This indicates that the simulation method can reliably predict the fracture behavior of materials under different strain conditions. The distribution of experimental and simulation data points in the forming limit diagram shows that materials exhibit different forming limits under varying strain conditions. Generally, at lower minor strain values, the major strain values are higher, indicating that the material has better ductility under low minor strain. As the minor strain increases, the major strain value decreases, indicating that the material becomes more prone to fracture. By comparing the results of the experimental and numerical simulations, the effectiveness of the J–C damage model in predicting forming limits is demonstrated, providing valuable theoretical guidance for further optimizing hot forming processes and material selection.

**Figure 31:**
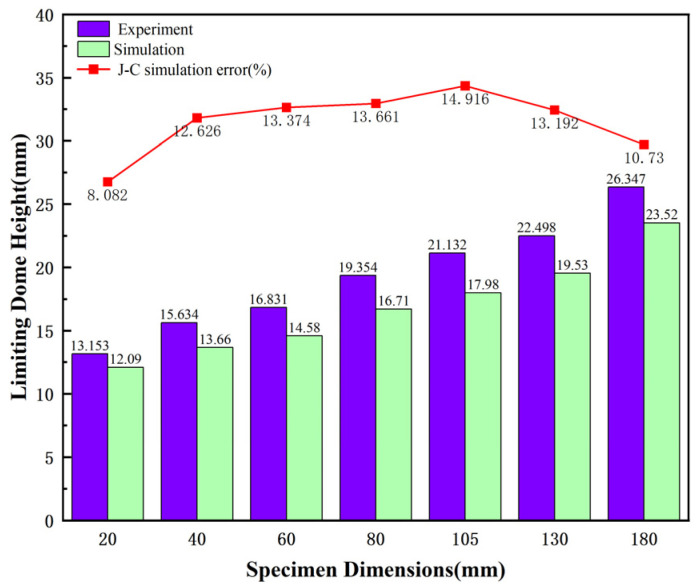
Comparison of the limiting dome height at the point of specimen failure.

**Figure 32:**
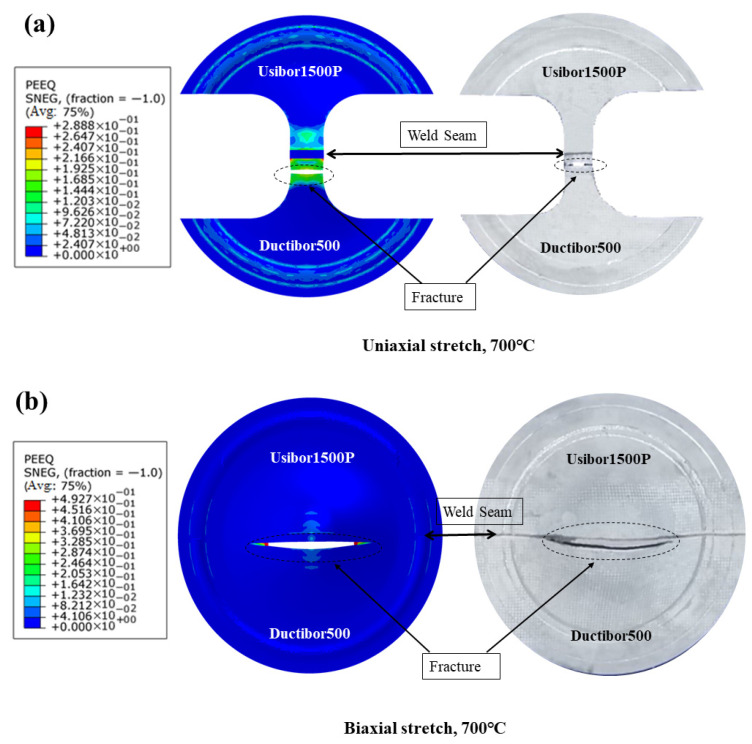
Comparison of fractured specimens from simulation and experimental tests: (**a**) uniaxial tension state; (**b**) biaxial tension state.

Figure 31 illustrates the limiting dome height (LDH) of the sample at the point of damage, comparing the simulation results with the experimental data. The simulation and experimental results show a close agreement. Figure 32 shows the limiting dome height (LDH) when the sample undergoes damage, with the maximum error between the simulation results and experimental data not exceeding 15%. The results indicate that the damage parameters determined in the J–C model through experiments accurately reflect the mechanical behavior during the hot forming process. They also offer more precise predictions for the fracture of TWBs.

Figure 32a,b show a comparison between the simulated and experimental fracture specimens of TWBs under uniaxial and biaxial stretching conditions. In both the experimental and simulation specimens, fracture occurred on the Ductibor500 base material side, with the fracture location closely matching the specimen. This indicates that the crack propagation path during the simulation process based on the J–C damage model is similar to that observed in the experimental process. Therefore, the hot Nakajima-type bulging experiments combined with the J–C damage model can accurately simulate the experimental forming limit testing process.

**Figure 33:**
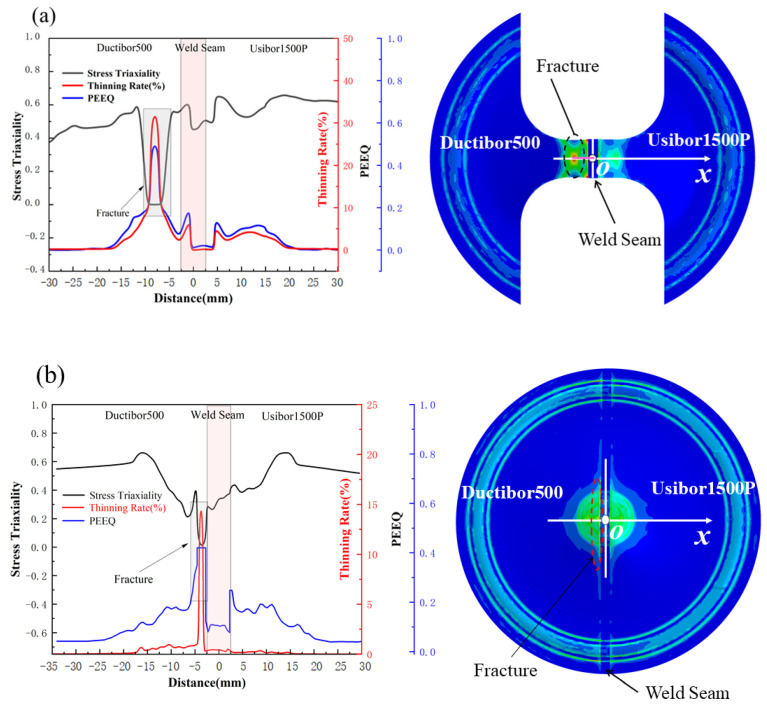
Distribution of stress triaxiality, PEEQ, and thinning rate along the *x*-axis direction at the point of fracture of the specimen: (**a**) uniaxial tension state; (**b**) biaxial tension state.

Figure 33 shows the distribution of stress triaxiality, PEEQ, and thinning rate along the *x*-axis direction at the point of fracture of the specimen. From the simulation results, it can be observed that the stress triaxiality in the *x*-axis direction for both specimen sizes is mainly below 0.6, which places this parameter within the ductile fracture sensitive zone. Notably, in the fracture region of the specimen, the stress triaxiality decreases significantly, while the PEEQ and thinning rate increase sharply. This indicates that the material undergoes substantial plastic deformation in this area, experiencing significant thinning. This local deformation, caused by stress concentration, makes this region the weak point for material fracture.

Due to the higher strength characteristics of the weld seam area, it exhibits a significant strain suppression effect during the bulging process, with its PEEQ consistently remaining at a lower level. The PEEQ is maintained between 0.04 and 0.08, which is more than 50% lower than that of the base material and stands in stark contrast to the damage evolution behavior in other regions. This indicates that the weld seam area plays a critical supporting role in the overall integrity of the structure. Its high strength characteristics help delay crack propagation and material failure.

The J–C damage model also demonstrates high prediction accuracy under low stress triaxiality conditions. This is primarily due to the model’s effective description of the material’s macroscopic mechanical behavior, particularly in cases where strain rate sensitivity and temperature effects are significant. The model is capable of accurately reflecting the material’s nonlinear dynamic response characteristics. 

## 6. Conclusions

This study uses the J–C damage model to investigate the damage mechanism of TWBs and weld seams with Usibor1500P and Ductibor500 as base materials. The model parameters were determined and calibrated through experiments and finite element simulations. The main conclusions are as follows:

(1) Through uniaxial tensile tests on notched specimens at room temperature, it was found that as the notch size decreases, stress concentration becomes more pronounced, resulting in higher stress triaxiality. This leads to an increase in the equivalent yield stress, while the equivalent fracture strain decreases, making the material more prone to fracture.
(2) By combining experimental testing and finite element simulation, the effects of temperature, strain rate, and stress triaxiality on fracture strain were analyzed. As stress triaxiality increases, the material’s fracture strain decreases, particularly in the 0~0.5 range, where stress triaxiality has a significant impact on material fracture failure. The lower the strain rate, the higher the fracture strain. Temperature also has a notable effect on the fracture failure strain of high-strength steel. As temperature increases, the material’s fracture strain shows an upward trend.
(3) A numerical model was constructed based on finite element analysis software, and the distribution characteristics of PEEQ and stress triaxiality parameters in the fracture region were extracted. The parameters were obtained, and the correlation coefficients for stress triaxiality, strain rate, and temperature were systematically calibrated. Ultimately, the J–C fracture criterion suitable for TWB weld seams, Usibor1500P high-strength steel, and Ductibor500 high-strength steel was established. The results show that the model can accurately predict the fracture locations and load–displacement curves for both smooth and notched specimens. The size of the notch and the level of stress triaxiality significantly influence the accuracy of the predictions. The J–C damage model demonstrates good applicability in predicting material fracture behavior under low stress triaxiality conditions.
(4) The parameters in the calibrated J–C damage model were further validated through hot Nakajima-type bulging tests. The model accurately predicts the fracture location in the TWB experimental hot forming process, with the simulated FLD showing good consistency with the experimental FLD. This suggests that the analysis based on the J–C damage model can effectively predict the fracture behavior of TWBs during the forming process, demonstrating strong potential for engineering applications.

## Figures and Tables

**Table 1:** Experimental plan for determining the J–C damage model parameters.

Experimental Design	Temperature (°C)	Strain Rate	Model Parameters
Shear Specimens	Room Temperature	0.001 s^−1^	
Smooth Specimens	Room Temperature	0.001 s^−1^	*D*_1_, *D*_2_, *D*_3_
Notched Specimens	Room Temperature	0.001 s^−1^	
Smooth Specimens	Room Temperature	0.01 s^−1^, 0.05 s^−1^, 0.1 s^−1^	*D*_4_
Smooth Specimens	600 °C, 700 °C, 800 °C	0.001 s^−1^	*D*_5_

**Table 2:** Chemical composition of Usibor1500P (wt%).

C	Si	Mn	Cr	Mo	P	S	Ti	Al	B
0.20–0.25	0.15–0.35	1.10–1.40	0.15–0.30	≤0.35	≤0.025	≤0.008	0.020–0.050	0.020–0.060	0.002–0.004

**Table 3:** Chemical composition of Ductibor500 (wt%).

C	Si	Mn	P	S	Ti	Nb	Al	B
≤0.10	≤0.50	≤1.90	≤0.030	≤0.025	≤0.150	≤0.090	>0.015	≤0.001

**Table 4:** Stress triaxiality and fracture strain of each specimen.

Specimen Type	Temperature (°C)	Strain Rate (s^−1^)	Usibor1500P		Weld Seam		Ductibor500	
			$\eta_{w}$	$\varepsilon_{f}$	$\eta_{w}$	$\varepsilon_{f}$	$\eta_{w}$	$\varepsilon_{f}$
Shear	20	0.001	0.055	0.624	0.051	0.663	0.081	0.6860
Smooth	20	0.001	0.334	0.3502	0.335	0.331	0.335	0.3305
R2	20	0.001	0.596	0.2512	0.559	0.263	0.583	0.2578
R5	20	0.001	0.575	0.2613	0.533	0.292	0.554	0.2646
R8	20	0.001	0.511	0.2793	0.511	0.303	0.501	0.2717

**Table 5:** J–C damage model parameters (*D*_1_, *D*_2_ and *D*_3_) of different regions.

Region	*D*_1_	*D*_2_	*D*_3_
Usibor1500P	0.1926	0.5253	−3.5847
Weld Seam	0.264	0.545	−6.1223
Ductibor500	0.24	0.7415	−6.2775

**Table 6:** Stress triaxiality and fracture strain of smooth samples under different strain rate conditions.

Specimen Type	Temperature (°C)	Strain Rate (s^−1^)	Usibor1500P		Weld Seam		Ductibor500	
			$\eta_{w}$	$\varepsilon_{f}$	$\eta_{w}$	$\varepsilon_{f}$	$\eta_{w}$	$\varepsilon_{f}$
Smooth	20	0.001	0.334	0.3502	0.335	0.331	0.335	0.3305
Smooth	20	0.01	0.334	0.2987	0.335	0.218	0.335	0.234
Smooth	20	0.05	0.334	0.2678	0.335	0.193	0.335	0.2033
Smooth	20	0.1	0.334	0.2475	0.335	0.181	0.335	0.188

**Table 7:** J–C damage model parameters (*D*_1_, *D*_2_, *D*_3_ and *D*_4_) of different regions.

Region	*D*_1_	*D*_2_	*D*_3_	*D*_4_
Usibor1500P	0.1926	0.5253	−3.5847	−0.063
Weld Seam	0.264	0.545	−6.1223	−0.109
Ductibor500	0.24	0.7415	−6.2775	−0.0996

**Table 8:** Stress triaxiality and fracture strain of smooth samples under different temperature conditions.

Specimen Type	Temperature (°C)	Strain Rate (s^−1^)	Usibor1500P		Weld Seam		Ductibor500	
			$\eta_{w}$	$\varepsilon_{f}$	$\eta_{w}$	$\varepsilon_{f}$	$\eta_{w}$	$\varepsilon_{f}$
Smooth	600	0.001	0.334	0.7018	0.335	0.346	0.335	0.5649
Smooth	700	0.001	0.334	0.8182	0.335	0.386	0.335	0.8560
Smooth	800	0.001	0.334	0.8828	0.335	0.513	0.335	1.2110

**Table 9:** J–C damage model parameters of different regions.

Region	*D*_1_	*D*_2_	*D*_3_	*D*_4_	*D*_5_
Usibor1500P	0.1926	0.5253	−3.5847	−0.063	2.914
Weld Seam	0.264	0.545	−6.1223	−0.109	0.596
Ductibor500	0.24	0.7415	−6.2775	−0.0996	3.907

**Table 10:** Thermophysical properties at various temperatures [38,39,40].

Temperature (°C)	20	200	400	600	800
Ductibor500					
Specific heat (J/kg/°C)	506	515	523	534	546
Conductivity (W/m/°C)	50	48	46	44	42
Expansion coefficient (μm/m/°C)	12.5	13.0	14.5	15.0	16.5
weld seam					
Specific heat (J/kg/°C)	460	500	550	600	650
Conductivity (W/m/°C)	35	30	25	20	15
Expansion coefficient (μm/m/°C)	12.0	12.5	13.0	13.5	14.0
Usibor1500P					
Specific heat (J/kg/°C)	481	493	502	511	522
Conductivity (W/m/°C)	45	43	41	39	37
Expansion coefficient (μm/m/°C)	11.4	12.6	13.0	14.5	15.5

## Data Availability

The original contributions presented in this study are included in the article. Further inquiries can be directed to the corresponding authors.

## References

[1] Šebestová H., Jambor M., Horník P., Novotný J., Mrňa L. (2024). Laser beam oscillation welding for fatigue properties enhancement of tailor-welded blanks. Thin-Walled Struct..

[2] Song Y., Yu C., Yu H., Zhao C. (2017). Mechanical Properties Improvement of Laser Tailor Welded Blanks of DP600 Steel by Magnetic Treatment. Metals.

[3] Merklein M., Johannes M., Lechner M., Kuppert A. (2014). A review on tailored blanks—Production, applications and evaluation. J. Mater. Process. Technol..

[4] Öztürk E., Arıkan H. (2023). Investigation of mechanical properties of laser welded dual-phase steels at macro and micro levels. Opt. Laser Technol..

[5] Mori K.I., Suzuki Y., Yokoo D., Nishikata M., Abe Y. (2020). Steel sheets partnered with quenchable sheet in hot stamping of tailor-welded blanks and its application to separation prevention of fractured components. Int. J. Adv. Manuf. Technol..

[6] Gautam V., Kumar A. (2019). Experimental and Numerical Studies on Formability of Tailor Welded Blanks of High Strength Steel. Procedia Manuf..

[7] Chen M.T., Cai A., Pandey M., Shen C., Zhang Y., Hu L. (2023). Mechanical properties of high strength steels and weld metals at arctic low temperatures. Thin-Walled Struct..

[8] Deepika D., Lakshmi A.A., Rao C.S., Sateesh N., Nookaraju B.C., Subbiah R. (2021). Formability of tailor welded blanks of aluminium alloy and steel—A review. Mater. Today Proc..

[9] Tang B., Wang Q., Wei Z., Meng X., Yuan Z. (2016). FE Simulation Models for Hot Stamping an Automobile Component with Tailor-Welded High-Strength Steels. J. Mater. Eng. Perform..

[10] Peister C., George R., Omer K., Worswick M.J., Malcolm S., Dykeman J., Yau C., Soldaat R., Bernert W. (2017). Forming of an axially tailored automotive channel section through hot stamping of tailor-welded blanks. J. Phys. Conf. Ser..

[11] Kinsey B., Liu Z., Cao J. (2000). A novel forming technology for tailor-welded blanks. J. Mater. Process. Technol..

[12] Raymond S.D., Wild P.M., Bayley C.J. (2004). On modeling of the weld line in finite element analyses of tailor-welded blank forming operations. J. Mater. Process. Technol..

[13] Tian H., Li Z., Deng X., Liu X. (2011). Investigation on the Elongation of Tailor Welded Blanks with Different Thickness. Adv. Mater. Res..

[14] Zhang J. (2007). Optimization of contact forces in tailor-welded blanks forming process. Int. J. Adv. Manuf. Technol..

[15] ChiuHuang C.K., Chiang M.F., Lee P.K. (2020). Numerical and Experimental Investigation on Hot Stamping of TWB B-Pillar. IOP Conference Series. Mater. Sci. Eng..

[16] Wang H., Liu L., Wang H., Zhou J. (2022). Control of defects in the deep drawing of tailor-welded blanks for complex-shape automotive panel. Int. J. Adv. Manuf. Technol..

[17] Ridha H., Thurner P.J. (2013). Finite element prediction with experimental validation of damage distribution in single trabeculae during three-point bending tests. J. Mech. Behav. Biomed. Mater..

[18] Johnson G.R., Cook W.H. (1985). Fracture characteristics of three metals subjected to various strains, strain rates, temperatures and pressures. Eng. Fract. Mech..

[19] Hu F., Liu X., Wang B., Xiang Y. (2024). Investigations on the Johnson-Cook Constitutive and Damage-Fracture Model Parameters of a Q345C Steel. Metals.

[20] Öztürk E., Cavusoglu O., Güral A. (2025). Experimental and Numerical Investigation of Strain Rate Dependent Flow and Fracture Behavior of 6181A-T4 Alloy Using the Johnson—Cook Model. Crystals.

[21] Pandya K.S., Roth C.C., Mohr D. (2020). Strain rate and temperature dependent fracture of aluminum alloy 7075: Experiments and neural network modeling. Int. J. Plast..

[22] Li X.Y., Zhang Z.H., Cheng X.W., Liu X.P., Zhang S.Z., He J.Y., Wang Q., Liu L.J. (2022). The investigation on Johnson-Cook model and dynamic mechanical behaviors of ultra-high strength steel M54. Mater. Sci. Eng. A.

[23] Skripnyak V.V., Skripnyak V.A. (2022). Mechanical Behavior of Alpha Titanium Alloys at High Strain Rates, Elevated Temperature, and under Stress Triaxiality. Metals.

[24] Wang Y., Zeng X., Chen H., Yang X., Wang F., Zeng L. (2021). Modified Johnson-Cook constitutive model of metallic materials under a wide range of temperatures and strain rates. Results Phys..

[25] Rajaraman D., Hertelé S., Fauconnier D. (2023). A novel calibration procedure of Johnson-Cook damage model parameters for simulation of scratch abrasion. Wear.

[26] Bal B.U., Karaveli K.K., Cetin B., Gumus B. (2019). The Precise Determination of the Johnson–Cook Material and Damage Model Parameters and Mechanical Properties of an Aluminum 7068-T651 Alloy. J. Eng. Mater. Technol..

[27] Zadpoor A.A., Sinke J., Benedictus R. (2011). 4-Numerical simulation modeling of tailor welded blank forming. In Woodhead Publishing Series in Welding and Other Joining Technologies. Tailor Welded Blanks Adv. Manuf..

[28] Zadpoor A.A., Sinke J., Benedictus R. (2021). Experimental and numerical studies on the influence of formability of AISI 316L tailor-welded blanks at different weld line orientations. J. Braz. Soc. Mech. Sci. Eng..

[29] Gopinath K., Narayanamurthy V., Khaderi S.N., Rao Y.V. (2024). Determination of Parameters for Johnson-Cook Dynamic Constitutive and Damage Models for E250 Structural Steel and Experimental Validations. J. Mater. Eng. Perform..

[30] Banerjee A., Dhar S., Acharyya S., Datta D., Nayak N. (2015). Determination of Johnson cook material and failure model constants and numerical modelling of Charpy impact test of armour steel. Mater. Sci. Eng. A.

[31] (2002). Metallic Materials—Tensile Testing at Room Temperature.

[32] Banerjee A., Dhar S., Acharyya S., Datta D., Nayak N. (2023). A Review of the Constitutive Modelling of Metals and Alloys in Machining Process. Arch. Comput. Methods Eng..

[33] Dou W., Xu Z., Hu H., Huang F. (2021). A generalized plasticity model incorporating stress state, strain rate and temperature effects. Int. J. Impact Eng..

[34] Choung J., Nam W., Lee D., Song C.Y. (2014). Failure strain formulation via average stress triaxiality of an EH36 high strength steel. Ocean Eng..

[35] (2009). Metallic Materials—Determination of Forming Limit Curves for Sheet and Strip—Part 2: Determination of Forming Limit Curves in the Laboratory.

[36] (2008). Metallic Materials-Sheet and Strip—Determination of Forming-Limit Curves—Part 2: Determination of Forming-Limit Curves in the Laboratory.

[37] Li Q., Li H., Gao S., Wang H., Wang G., Sun Y., Gu D., Wu Y. (2025). Study on hot formability of high-strength steel laser tailor welded blanks based on microscopic damage modeling. Int. J. Adv. Manuf. Technol..

[38] Li Q., Li H., Gao S., Wang H., Wang G., Sun Y., Gu D., Wu Y. (2020). Elevated temperature material properties of advanced high strength steel alloys. J. Constr. Steel Res..

[39] Maraveas C., Fasoulakis Z.C., Tsavdaridis K.D. (2017). Mechanical properties of High and Very High Steel at elevated temperatures and after cooling down. Fire Sci. Rev..

[40] Keränen L., Kangaspuoskari M., Niskanen J. (2021). Ultrahigh-strength steels at elevated temperatures. J. Constr. Steel Res..

